# The Histopathological Types and Distribution Characteristics of Gastric Mixed Tumors

**DOI:** 10.3389/fonc.2022.873005

**Published:** 2022-06-17

**Authors:** Fang-Heng Zhu, Yang-Kun Wang, Jun-Ling Zhou, Nian-Long Meng, Yue Wang, Bo Jiang, Su-Nan Wang

**Affiliations:** ^1^ Department of Pathology, Xinxiang Central Hospital, Xinxiang, China; ^2^ Department of Pathology, Foresea Life Insurance Guangzhou General Hospital, Guangzhou, China; ^3^ Shenzhen Nanshan District People’s Hospital, Shenzhen, China; ^4^ Department of Pathology, The 989th Hospital of the Joint Logistics Support Force of The Chinese People’s Liberation Army, Luoyang, China; ^5^ Shenzhen Hezheng Hospital, Shenzhen, China; ^6^ Department of Pathology, People’s Liberation Army Joint Logistic Support Force 990th Hospital, Zhumadian, China; ^7^ Shenzhen Polytechnic, Shenzhen, China

**Keywords:** gastric tumor, mixed tumor, mixed tissue type, mixed organization, gene target type

## Abstract

**Objective:**

The present study aimed to investigate the histopathological types and distribution characteristics of gastric mixed tumors.

**Methods:**

Detailed histological observations, together with related immunohistochemical and genetic tests, were analyzed on 960 surgically resected samples in 6 hospitals with gastric mixed tumors from May 2017 to May 2021 in this retrospective study.

**Results:**

Epithelial-derived tumors accounted for 80.10% (769/960) of the gastric mixed tumor samples studied, and tumors of different tissue origins accounting for 10.83% (104/960), mesenchymal-derived tumors accounting for 6.25% (60/960), neuroendocrine tumors accounting for 2.40% (23/960), and lymphoma accounting for 0.42% (4/960). The histological types of gastric mixed tumors identified as most commonly were epithelial originated, followed by mixed tumors of different tissue originated, then mixed neuroendocrine, lymphoma, and mesenchymal originated in sequence. The histological number of gastric mixed tumors was ≤ 3 in 83.23% (799/960) of cases and > 4 in 16.77% (161/960) of cases. The mixed histological patterns of gastric mixed tumors were divided into three types: those with tumor cells interspersed with each other, those with incomplete fibrous tissue separation, and those without fibrous tissue separation. The gene target characteristics of gastric mixed tumors were the existence of multi-gene mutation, including human epidermalgrowth factor receptor-2 (HER2) gene amplification, key result areas (K-ras) and platelet-derived growth factor receptor alpha (PDGFRA).

**Conclusion:**

Gastric mixed tumors should be adequately sampled, each piece of tissue should be involved in the morphological proportional division of the tumor, and any independent histological component should be written into the pathological examination report.

## Introduction

Gastric tumors are highly heterogeneous, which is reflected in their occurrence, recurrence, metastasis, morphology, immunophenotype, DNA ploidy, molecular biology, and genetics (1–4). In 1965, Lauren classified gastric tumors into three types: intestinal, diffused, and mixed (5), and in 2010 and 2019, the classification of gastric mixed adenocarcinoma was proposed in the World Health Organization (WHO) classification of gastrointestinal cancers (6, 7). The WHO classification and the Lauren’s classification are commonly used in current clinicopathological diagnosis. However, the description of more than two histological types in pathological reports varies greatly, and pathological diagnoses are not identical.

Most pathological reports are written for cases when mixed components is equal to or greater than 30%, while cases with mixed components lower than 30% are ignored. In addition, only major components and minor components are distinguished, and the histological types of various mixed components are not included (8). A previous study found that lymph node metastasis can still occur in the minor part of a gastric tumor of < 10% and the metastasis is the histomorphological change of < 10% (9). A pathological examination report—especially the one including any poorly differentiated tumors—is more conducive to the study of the characteristics, metastasis of recurrent tumors and the accurate treatment of primary mixed gastric tumors, which should be used for accurate treatment (10).

In order to standardize the pathological diagnosis of gastric mixed tumors, the present study collected 960 cases of gastric mixed tumors from the pathology departments of six hospitals and analyzed their characteristics. The distribution, number of samples, histopathological characteristics, differential diagnosis, and writing of the pathological diagnosis reports were examined and identified in order to provide quantitative reference indicators for evaluating the prognosis of gastric mixed tumors and the benefits of targeted drug therapy.

## Materials and Methods

### Subjects

A total of 3,946 cases with surgically removed gastric tumors or tumors of the esophagogastric junction from the Foresea Life Insurance Guangzhou General Hospital, Xinxiang Central Hospital, Shenzhen Nanshan District People’s Hospital, The 989th Hospital of the Joint Logistics Support Force of the Chinese People’s Liberation Army, Shenzhen Hyzen Hospital, and People’s Liberation Army Joint Logistic Support Force 990th Hospital, between May 2017 and May 2021 were retrospectively analyzed. Among these cases, 960 were of mixed gastric tumors. Ages of these patients included in the study ranged from 11 to 94 years, with an average age of 58.7 years.

This study was approved by the Ethics Committee of Luoyang 150 Central Hospital (No.132102310008). Written informed consent was obtained from the participants.

### Methods

Within 30 minutes after surgery, all specimens were fixed with 10% fresh neutral buffered formalin solution for 8–48 hours, with a volume ratio of fixing solution to tissue of 10:1. The tissue in the tumor area was fully sampled, and conventional sampling was conducted according to the color, texture, and depth of invasion. If the tumor diameter was less than 3 cm, all tumors and their surrounding area were removed. For gastric tumors equal to or greater than 4 cm in diameter, 10–15 pieces of tissue were removed, with no less than 4 pieces of tissue taken from the junction between the tumor and normal gastric tissue. One piece of tissue was taken from the deepest infiltration point and one was taken from the nearest serous layer. The size of tissue removed was 2 cm × 1.5 cm × 0.3 cm.

Each tissue block was included in the proportional division of tumor morphology. In addition, one piece of proximal tissue and one piece of distal tissue from the cutting edge were included. All lymph nodes and cancerous nodules were cut into sections. Hematoxylin and eosin staining, light microscopy, immunohistochemical staining, and gene detection were then conducted.

### Pathological Classification

Following the WHO Histological Classification of Gastric tumor in Digestive System Tumors (2019 edition) (7) and Gastric Tumor Pathology (2019 edition) (8), the lesions were divided into five types: gastric mixed adenocarcinoma, mixed neuroendocrine carcinoma, mixed lymphoma, mixed mesenchymal cancer, and mixed cancer of different histological origin.

### Immunohistochemical Staining

All immunohistochemical reagents and working solutions were purchased from Shenzhen Dartmon Biotechnology Co., Ltd. (China). All procedures were conducted according to the kit instructions.

### Drug Targets and Genetic Testing

Detections of the human epidermalgrowth factor receptor-2 (HER2), EGFR, key result areas (K-ras) genes and programmed cell death-1 (PD-1)/programmed cell death-Ligand 1 (PD-L1) (Estimated Glomerular Filtration Rate, EGFR)were conducted for gastric tumors of epithelial origin. The reagents, probe, and procedures of fluorescence in situ hybridization (FISH) were referred to literature (11, 12). Pyrophosphate sequencing was adopted for the quantitative detection of K-ras gene mutation (13). The multiple endocnne neoplasia 1 (MEN1) gene was mainly detected in gastric neuroendocrine tumors, the Akt and c-Myc genes were mainly detected in gastric lymphoma, and the platelet-derived growth factor receptor alpha (PDGFRA) and c-kit genes were mainly detected in gastric mesenchymal tumors.

### Statistical Methods

Normally distributed measurement data were expressed as mean ± standard deviation (SD) and the categorical data were expressed as n(%).

## Results

### Clinical Features

A total of 571 males and 389 females were included in this study, and the number of male patients was higher than that of female patients in all stages. The age of onset in 62.81% (603/960) of patients was ≤ 60 years, and 37.19% (357/960) patients were > 60 years old. The number of patients aged ≤ 60 years was higher than that of those aged > 60 years, but with no statistical difference. The number of histological types was ≤ 3 in 83.23% (799/960) of patients and > 4 in 16.77% (161/960) patients. The gene target types were ≤ 3 in 87.60% (841/960) of patients and > 4 in 12.40% (119/960) of patients (see **Table 1**).

**Table 1:** Analysis of clinicopathological parameters of gastric mixed tumors.

Histological origin	Types	n (%)	Male/Female	Age of onset		Number of histological types		Gene target type	
				≤60	>60	≤3	>4	≤3	>4
Epithelial neoplasm	Gastric mixed epithelial neoplasm	769 (80.1)	461/308	515 (67.0)	254 (33.0)	622 (80.9)	147 (19.1)	665 (86.5)	104 (13.5)
	Common type of mixed adenocarcinoma	534 (55.6)	325/209	356 (66.7)	178 (33.3)	426 (79.8)	108 (20.2)	447 (83.7)	87 (16.3)
	Infrequent type of mixed adenocarcinoma	194 (20.2)	112/82	133 (68.6)	61 (31.4)	160 (82.5)	34 (17.5)	180 (92.8)	14 (7.2)
	Rare type of mixed adenocarcinoma	19 (2.0)	11/8	12 (63.2)	7 (36.8)	17 (89.5)	2 (10.5)	18 (94.7)	1 (5.3)
	Gastric mixed adenosquamous carcinoma	22 (2.3)	13/9	14 (63.6)	8 (36.7)	19 (86.4)	3 (13.6)	20 (90.9)	2 (9.1)
Neuroendocrine	Gastric mixed neuroendocrine neoplasm	23 (2.4)	14/9	8 (34.8)	15 (65.2)	23 (100.0)	0 (0.0)	23 (100.0)	0 (0.0)
	Mixed two types of the neuroendocrine tumor G1/G2/G3	8 (0.8)	5/3	2 (25.0)	6 (75.05)	8 (100.0)	0 (0.0)	8 (100.0)	0 (0.0)
	Mixed neuroendocrine large cell carcinoma - small cell carcinoma	3 (0.3)	2/1	1 (33.3)	2 (66.7)	3 (100.0)	0 (0.0)	3 (100.0)	0 (0.0)
	Neuroendocrine carcinoma-mixed Intreroneural neoplasms	12 (1.3)	7/5	5 (41.7)	7 (58.3)	12 (100.0)	0 (0.0)	12 (100.0)	0 (0.0)
Lymphoma	Gastric mixed lymphoma	4 (0.4)	3/1	0 (0.0)	4 (100.0)	4 (00.0)	0 (0.0)	4 (00.0)	0 (0.0)
	Mixed diffused large B cell lymphoma- MALT lymphoma	2 (0.2)	1/1	0 (0.0)	2 (100.0)	2 (100.0)	0 (0. 0)	2 (100.0)	0 (0. 0)
	Mixed diffused large B cell lymphoma-Mantle cell lymphoma	1 (0.1)	1/0	0 (0.0)	1 (100.0)	1 (100.0)	0 (0.0)	1 (100.0)	0 (0.0)
	Mixed MALT lymphoma-Burkitt lymphoma	1 (0.1)	1/0	0 (0.0)	1 (100.0)	1 (100.0)	0 (0.0)	1 (100.0)	0 (0.0)
Mesenchymal tissue	Gastric mixed mesenchymal neoplasm	60 (6.3)	34/26	28 (46.7)	32 (53.3)	51 (85.0)	9 (15.0)	60 (100.0)	0 (0.0)
	Common type of mixed mesenchymal tissue	42 (4.4)	24/18	20 (47.6)	22 (52.4)	35 (83.35)	7 (16.7)	42 (100.0)	0 (0.0)
	Infrequent type of mixed mesenchymal tissue	16 (1.7)	9/7	7 (43.8)	9 (56.3)	14 (87.5)	2 (12.5)	16 (100.0)	0 (0.0)
	Rare type of mixed mesenchymal tissue	2 (0.2)	1/1	1 (50.0)	1 (50.0)	2 (100.0)	0 (0.0)	2 (100.0)	0 (0.0)
Different tissue sources	Mixed neoplasms of different tissue origins	104 (10.8)	59/45	52 (50.0)	52 (50.0)	99 (95.2)	5 (4.8)	89 (85.6)	15 (14.4)
	Adenocarcinoma-Neuroendocrine tumor	54 (5.6)	30/24	29 (53.7)	25 (46.3)	51 (94.45)	3 (5.6)	49 (90.7)	5 (9.3)
	Adenocarcinoma-Mesenchymal tumor	31 (3.2)	18/13	17 (54.8)	14 (45.2)	29 (93.5)2	(6.5)	24 (77.4)	7 (22.6)
	Adenocarcinoma-Tumors of the lymphatic hematopoietic system	9 (0.9)	5/4	4 (44.4)	5 (55.6)	9 (100.0)	0 (0.0)	6 (66.7)	3 (33.3)
	Neuroendocrine-Mesenchymal tumor	8 (0.8)	5/3	2 (25.0)	6 (75.0)	8 (100.0)	0 (0.0)	0 (100.0)	0 (0.0)
	Neuroendocrine-Tumors of the lymphatic hematopoietic system	2 (0.2)	1/1	0 (0.0)	2 (100.0)	2 (100.0)	0 (0.0)	0 (100.0)	0 (0.0)

### The Type and Distribution of Mixed Gastric Tumors

**Figure 1:**
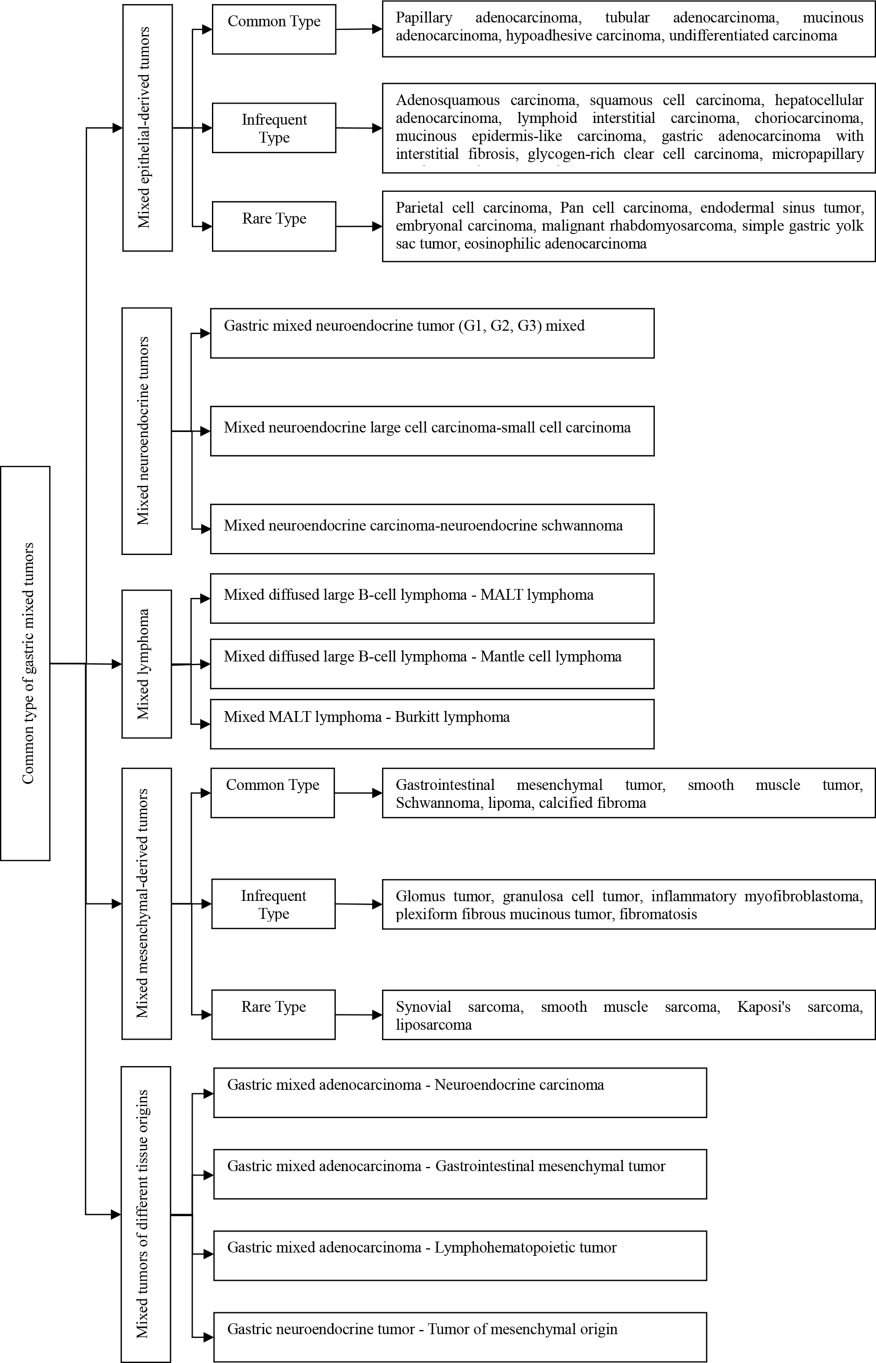
Common types of gastric mixed tumors.

**Figure 2:**
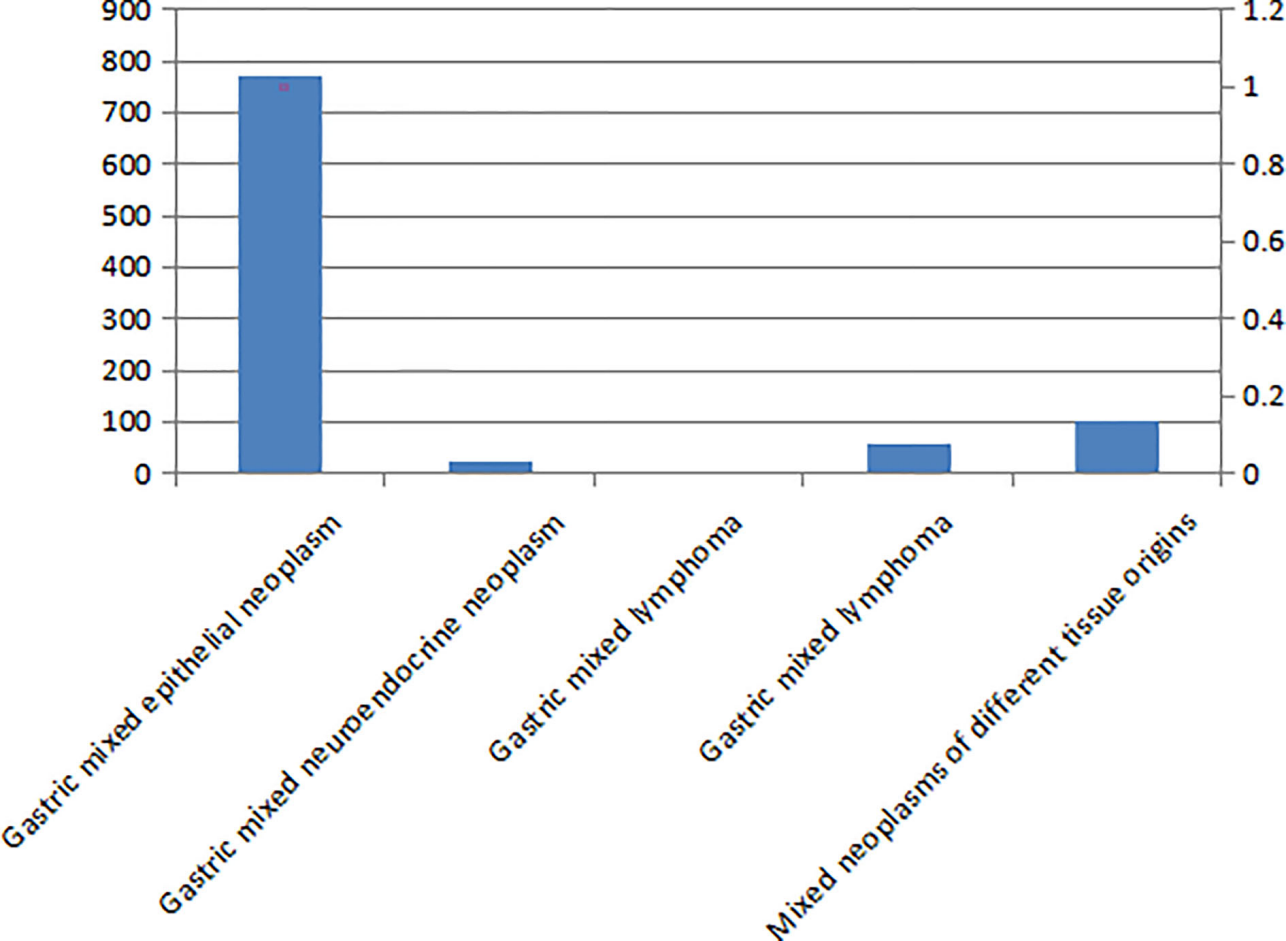
Common distribution of gastric mixed tumors.

There are five types of mixed gastric tumors (see **Figure 1**). The distribution of these tumors identified in the study is shown in **Figure 2**.

### The Histological Features of Mixed Gastric Tumors

**Figure 3:**
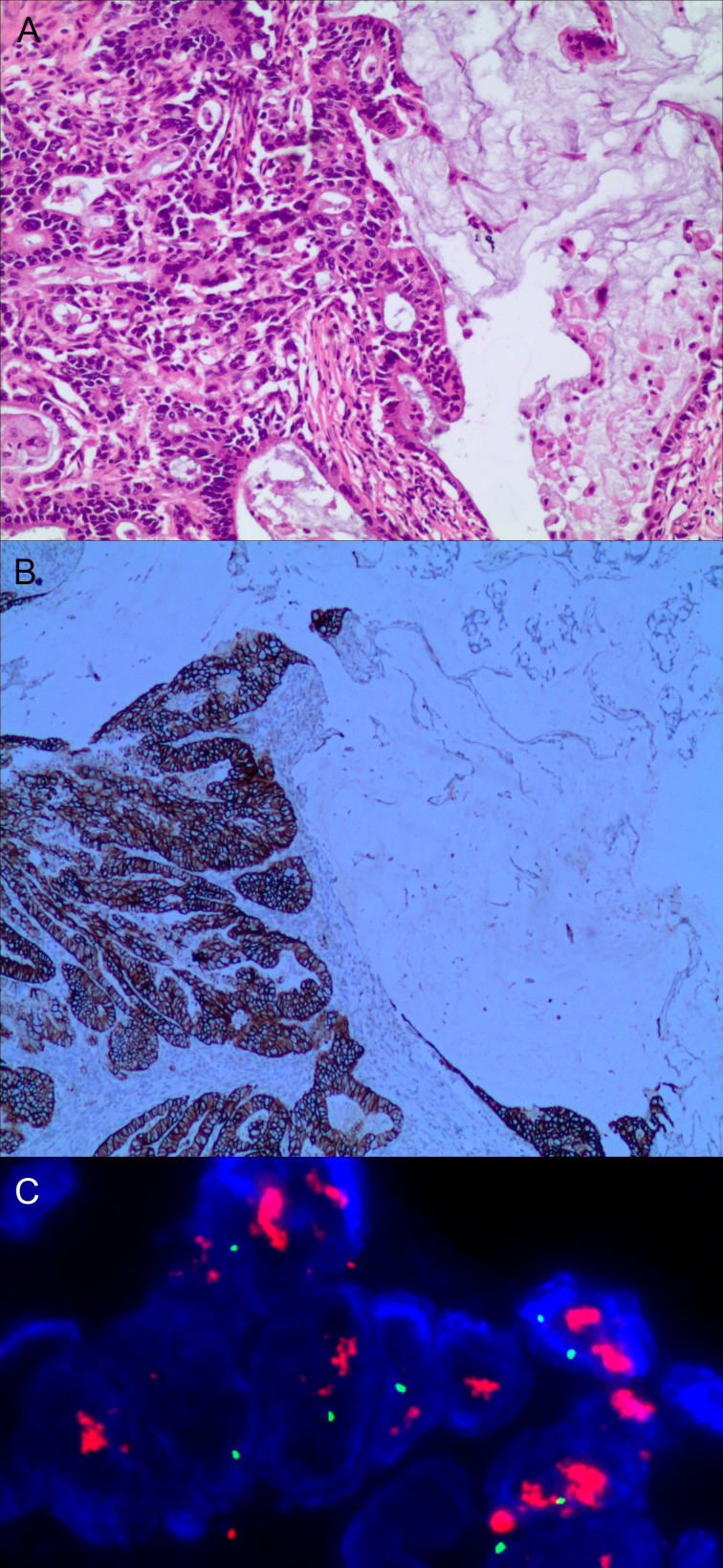
Gastric mixed adenocarcinoma. **(A)** Mixed mucinous adenocarcinoma-papillary adenocarcinoma (70% mucinous adenocarcinoma/30% papillary adenocarcinoma), hematoxylin and eosin staining × 100. **(B)** HER2 protein expression. Papillary adenocarcinoma was partially 3+ positive. Mucinous adenocarcinoma was partially negative. En Vision method × 200. **(C)** FISH tests, papillary adenocarcinoma cluster amplification. Red represents the probe signal; green represents chromosome 17. FISH, fluorescence *in situ* hybridization; HER2: human epidermalgrowth factor receptor-2.

**Figure 4:**
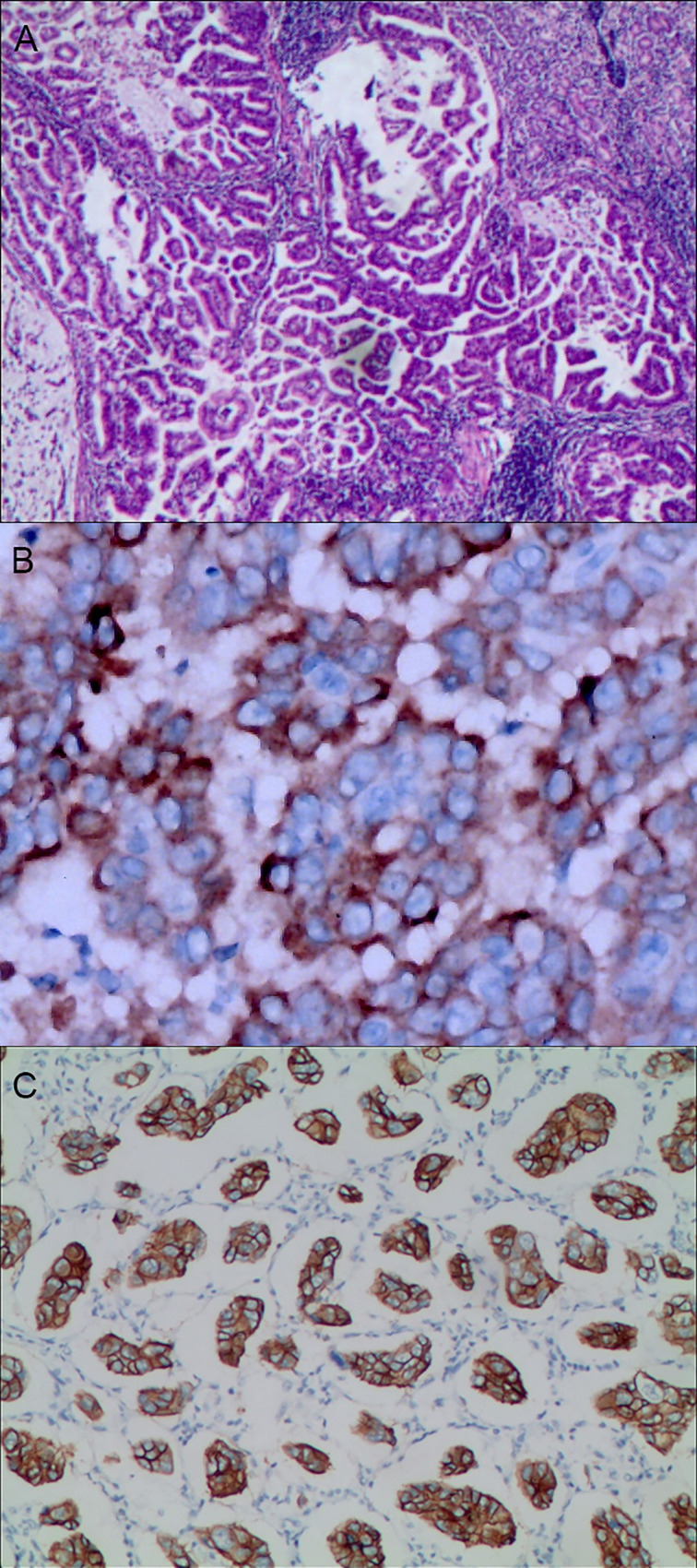
Gastric mixed adenocarcinoma. **(A)** Gastric mixed tubular adenocarcinoma-micropapillary carcinoma (20% tubular adenocarcinoma/80% micropapillary carcinoma), hematoxylin and eosin staining × 100. **(B)** Positive MUC1, gastric invasive micropapillary carcinoma. The micropapillary carcinoma cells were clustered with an irregular central cavity and a surrounding space with the stroma. En Vision method × 400. **(C)** Positive EMA. The micropapillary carcinoma cells were reversed poleward, presenting an inside-out shape. En Vision method × 200. MUC1: bovine mucin 1.

A total of 80.10% (769/960) of patients had gastric mixed tumors of epithelial origin, which were classified into four types: common, infrequent, rare, and adenosquamous. The common type included papillary adenocarcinoma, tubular adenocarcinoma, mucinous adenocarcinoma, and low adhesion carcinoma (see **Figure 3A**). The infrequent type was mainly composed of squamous carcinoma, hepatoid adenocarcinoma, lymphoid stromal carcinoma, undifferentiated carcinoma, choriocarcinoma, mucoepidermoid carcinoma, stomach adenocarcinoma with stromal fibrosis, clear-cell carcinoma rich in glycogen, micropapillary carcinoma (see **Figure 4A**), columnar cell mucinous carcinoma, and ulcerative carcinoma. The rare type included parietal cell carcinoma, Paneth cell carcinoma, endodermal sinus tumor, embryonal carcinoma, malignant rhabdoid tumor, simple gastric yolk sac tumor, and eosinophilic adenocarcinoma, accounting for 2.50% (24/960).

A total of 0.73% (7/960) of all samples studied were gastric mixed gonadal squamous carcinoma, 2.40% (23/960) were gastric mixed neuroendocrine tumors, and 0.42% (4/960) were gastric mixed lymphoma, including diffused large B-cell lymphoma-MALT lymphoma mixed, diffused large B-cell lymphoma-mantle cell lymphoma mixed, and MALT lymphoma-Burkitt lymphoma mixed.

**Figure 5:**
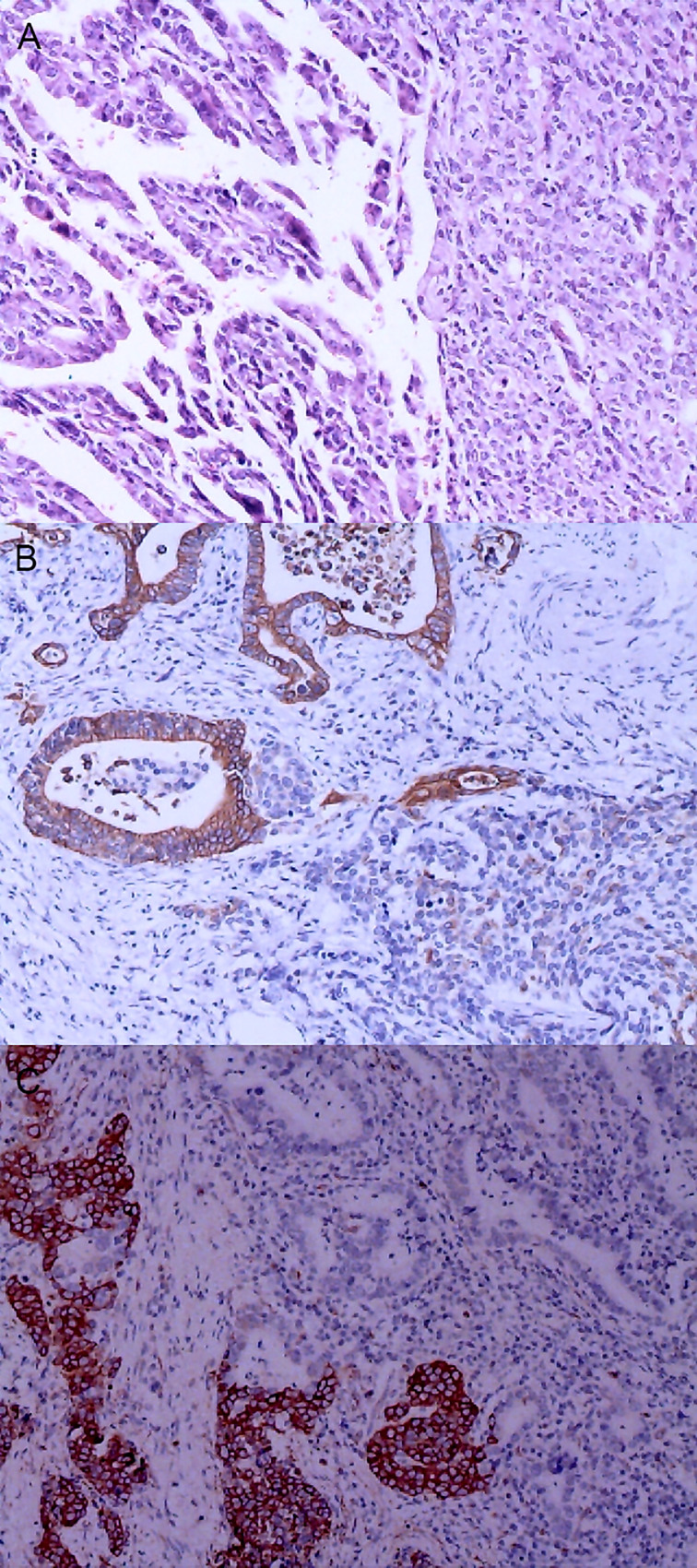
Gastric mixed carcinoma. **(A)** Gastric mixed papillary adenocarcinoma-neuroendocrine tumor (70% papillary adenocarcinoma/30% neuroendocrine tumor, G1), hematoxylin and eosin staining × 40. **(B)** Positive CgA. CgA was negative in adenocarcinoma components and positive in carcinoid components. En Vision method ×200. **(C)** CKpan was negative in carcinoid components and positive in adenocarcinoma components. En Vision method × 200.

**Figure 6:**
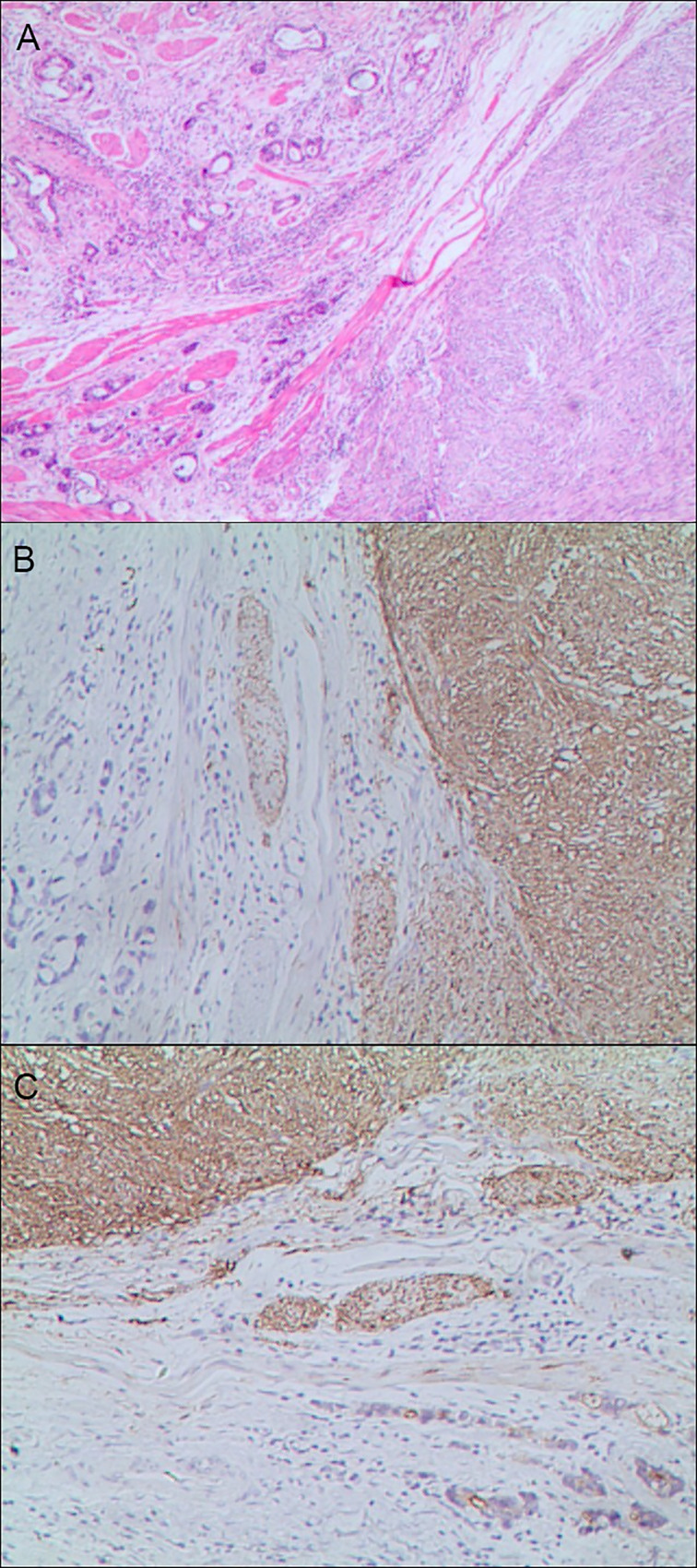
Gastric mixed tumors. **(A)** Gastric mixed gastrointestinal stromal tumor with low nuclear fission rate (80% tubular adenocarcinoma with moderately differentiated gastrointestinal stromal tumor/20% tubular adenocarcinoma), hematoxylin and eosin staining × 20. **(B)** Immunohistochemical staining of gastrointestinal stromal tumor showed CD117 was partially positive. En Vision method × 20. **(C)**. Gastrointestinal stromal tumor showed DOG1 was partially positive. En Vision method × 20.

Gastric mixed mesenchymal tumors accounted for 6.25% (60/960). This category was classified into common, infrequent and rare mixed mesenchymal tissues. Mixed tumors of different tissue origin accounted for 10.83% (104/960), including adenocarcino-neuroendocrine tumors (see **Figure 5A**), adenocarcino-mesenchymal tumors (see **Figure 6A**), adenocarcino-lymphohematopoietic system tumors, neuroendocrine-mesenchymal tumors, and neuroendocrine-lymphohematopoietic system tumors (see **Table 1**).

### HER2 Protein Expression and Other Immunophenotypes in Mixed Gastric Tumors

The positive expression of HER2 protein was localized in the cell membrane. HER2 protein was expressed in mixed mucinous adenocarcinoma-papillary adenocarcinoma (see **Figure 3B**). In gastric mixed tubular adenocarcinoma-micropapillary carcinoma, bovine mucin 1 (MUC1) marked positive with an irregular central cavity, surrounded by a space with stroma (see **Figure 4B**); positive EMA. The micropapillary tumor masses were poleward reversed, presenting an inside-out shape (see **Figure 4C**). In gastric mixed papillary adenocarcinoma-neuroendocrine tumors, there was negative expression of CgA-labeled adenocarcinoma components. Positive expression of neuroendocrine tumor by En Vision Method (9) (**Figure 5B**); the CKpan expression was negative for neuroendocrine components and positive for adenocarcinoma components by En Vision method (see **Figure 5C**). Gastric mixed gastrointestinal stromal tumor with low nuclear fission rate-moderately differentiated tubular adenocarcinoma. CD117 and DOG1 were partially positive in gastrointestinal stromal tumors (see **Figures 6B, C**, respectively). In gastric mixed gastrointestinal stromal tumor-hemangiomas, there was a boundary between the gastrointestinal stromal tumor and the hemangioma.

### Genetic Test Results of Mixed Gastric Tumors

The characteristics of FISH gene amplification detection were as follows: cluster amplification (see **Figure 3C**), large granular amplification, and dot amplification (see **Table 1**).

### Histological Pattern of Mixed Gastric Tumors

There were three types of mixed gastric tumor: the type that tumor cells were interspersed with each other, the type with incomplete fibrous tissue separation and the type without fibrous tissue separation.

## Discussion

The histological type of gastric tumor is an important factor affecting prognosis, as well as an important basis for determining the range of surgical resection and formulating a reasonable surgical plan (14). Similarly, histological type and differentiation grade of mixed gastric tumors were also important prognosic factors, The efficacy of neoadjuvant chemotherapy, and the prognostic indicators (15). There are significant differences in biological characteristics, degree of malignancy, pathological features, and prognosis based on the accurate classification and diagnosis of mixed gastric tumors (16, 17). At present, the pathological diagnostic criteria for gastric mixed tumors are not unified, which affects clinical treatment and prognosis. Furthermore, with the emerging of deepening biological research, new molecular targeted therapy drugs, accurate pathological diagnosis of gastric tumors with large heterogeneity were important (18, 19).

There are several problems with current pathological diagnosis reports for mixed gastric tumors. First, they are inconsistent. Some researchers have suggested the classification of a tumor as mixed tumor if the mixed composition is ≥ 30%, with the adoption of the XX tumor if the mixed composition is < 30% (20). Others have suggested that mixed gastric tumors should be diagnosed according to their major and minor components, such as XX cancer with XX differentiation (21). Second, the number of pathological samples of mixed gastric tumors varies greatly (22–25).

Based on the histopathological analysis of 960 cases of gastric mixed tumors, the present study suggests that the following key sampling points should be adopted for gastric mixed tumors: (1) the tissue of tumor area should be fully sampled, and conventional sampling should be conducted according to the color, texture, and depth of invasion; (2) if the tumor diameter is less than 3 cm, all tumor samples should be collected, including the tumor and surrounding normal tissue; (3) if the tumor diameter is equal to or greater than 4 cm, 10–15 sections should be removed from each tumor, and no less than 4 pieces of tissue should be taken from the junction between the tumor and the normal stomach tissue; (4) the size of sampled tissue should be 2 cm × 1.5 cm × 0.3 cm; (5) each tissue block should be involved in the proportional division of tumor morphology; (6) one piece of tissue from the proximal edge and one from the distal edge are required; (7) two pieces of tissue involving the deepest infiltration point and the nearest serous membrane layer should be included.

In the present study, all lymph nodes and cancerous nodules were excised in sections. The pathological examination report showed that all tissue samples removed were involved in the proportional division of tumor morphology, and each tissue was calculated by area. The findings of this study indicate that each independent histological component should be written into the pathology report in proportion to area. Any poorly differentiated parts, regardless of lesion size of the mixed components, might be important risk factors affecting prognosis.

In patients with advanced gastric tumors receiving neoadjuvant chemotherapy, primary lesion assessment based on histology has been found to be independently correlated with prognosis (26). In the present study, it was suggested that only with a complete report making clinical implementation of effective individualized treatment be possible. Adequate sampling of mixed gastric tumors should be used for molecular detection in different regions, thereby providing quantitative reference indicators for the benefits of targeted antineoplastic drug therapy. Furthermore, detailed clinicopathological reports describing the percentage of each histological type and degree of differentiation of mixed gastric tumors might be significant for accurate prognostic assessment. Moreover, with the development and wide use of endoscopic biopsies and endoscopic ultrasound fin-needle biopsy, preoperative diagnosis of these mixed tumor patients might be helpful for selection of treatment strategy (27, 28).

Mixed gastric tumors consist of two or more histological components within a tumor, each of which has an independent histological structure. There are many reports on mixed gastric tumors, but most of them are individual case reports, and there is still a lack of large sample studies (29–31). In the present study, a large sample of 960 patients with gastric mixed tumors was collected and analyzed. The mixed histological patterns of gastric mixed tumors were divided into three types: those with tumor cells interspersed with each other, those with incomplete fibrous tissue separation, and those without fibrous tissue separation. The histological number of mixed gastric tumors ≤ 3 accounted for 83.2% of cases, while those > 4 accounted for 16.8%. Usually two to three tissues were mixed, with as many as six or more tissues mixed. The gene targets of mixed gastric tumors were found to include HER2 gene amplification, K-ras and PDGFR. The distribution characteristics of gastric mixed tumors were identified as most commonly of epithelial origin, followed by mixed tumors of different tissue origin, with fewer mixed neuroendocrine tumors, lymphoma, and tumors of mesenchymal origin. At present, the origin of the mixed tumors is not very clear, but there are two widely accepted hypotheses (32, 33): (1) those originating from different cell lines; (2) those derived from endodermal pluripotent stem cells, which are the result of the pluripotent differentiation of stem cells during tumor development.

There were also several limitations in this study. First, there was unavoidable biases in this study due to its retrospective nature. Secondly, findings in this study should be verified in the future, especially during preoperative time.

## Conclusions

The present study proposed an effective method of sampling mixed gastric tumors. If the tumor diameter is less than 3 cm, all tumors and their surrounding areas should be removed. For gastric tumors with a diameter greater than 4 cm, 10–15 pieces of tissue should be removed from each, and no fewer than four pieces of tissue should be taken from the junction between the tumor and the normal gastric tissue. Any independent histological component of mixed gastric tumors should be included in the pathological examination report. Only when the test report is complete is the clinical implementation of effective individualized treatment possible.

## Data Availability Statement

The original contributions presented in the study are included in the article/supplementary material. Further inquiries can be directed to the corresponding author.

## Ethics Statement

The studies involving human participants were reviewed and approved by Ethics Committee of Luoyang 150 Central Hospital. The patients/participants provided their written informed consent to participate in this study.

## Author Contributions

Conception and design of the research: Y-KW, S-NW, and F-HZ. Acquisition of data: N-LM. Analysis and interpretation of the data: YW and BJ. Statistical analysis: J-LZ. Writing of the manuscript: S-NW and F-HZ. Critical revision of the manuscript for intellectual content: Y-KW. All authors read and approved the final draft.

## Funding

Supported by the Key Scientific and Technological Research Project of Henan Province (No. 132102310008).

## Conflict of Interest

The authors declare that the research was conducted in the absence of any commercial or financial relationships that could be construed as a potential conflict of interest.

## Publisher’s Note

All claims expressed in this article are solely those of the authors and do not necessarily represent those of their affiliated organizations, or those of the publisher, the editors and the reviewers. Any product that may be evaluated in this article, or claim that may be made by its manufacturer, is not guaranteed or endorsed by the publisher.
